# Granulomatous Mastitis

**DOI:** 10.1590/0037-8682-0452-2021

**Published:** 2021-11-12

**Authors:** Fatma Kesmez Can, Fadime Güven, Erdem Karadeniz

**Affiliations:** 1 Ataturk University, Medical Faculty, Department of Infectious Diseases and Clinical Microbiology, Erzurum, Turkey.; 2 Ataturk University, Medical Faculty, Department of Radiology, Erzurum, Turkey.; 3 Ataturk University, Medical Faculty, Department of General Surgery, Erzurum, Turkey.

**FIGURE 1:**
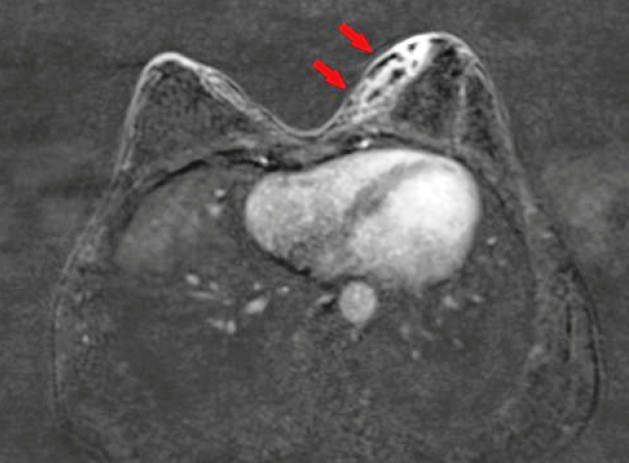
Axial T1 weighted MRI image: Pre-treatment image shows fistulized thick-walled abscess formation with thickening of the skin in the lower inner quadrant of the left breast (arrows).

**FIGURE 2:**
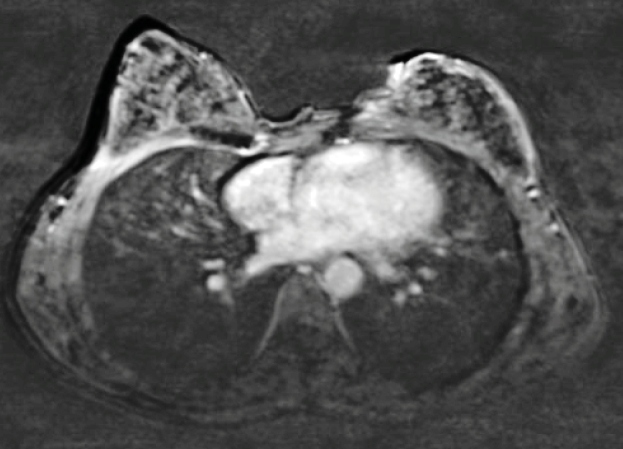
Axial T1 weighted MRI image: Post-treatment axial contrast-enhanced image shows no inflammation and/or abscess at the left breast.

A 22-year-old woman with no history of pregnancy or comorbidities was admitted to the Infectious Diseases Clinic at Atatürk University Medical Faculty Hospital with a 2-month history of swelling, pain, and purulence in the left breast. Her readings were as follows: leukocyte count, 7300; neutrophil value, 63%; platelet count, 232,000; C-reactive protein, 3; sedimentation rate, 5; aspartate aminotransferase, 26; alanine aminotransferase 24, PPD, 25 mm; and Quantiferon was positive. The patient was diagnosed with pulmonary tuberculosis. Ultrasonography revealed a dense lesion in the lower left breast. Lymphadenopathy (18×7 mm) was observed in the left axillary region^1^. Despite 2 weeks of antibiotic therapy, the magnetic resonance imaging (MRI) of the breast revealed a fistulized, thick-walled abscess formation on the skin, accompanied by skin thickening in the lower inner quadrant of the left breast (Figure 1). Biopsy results indicated granulomatous mastitis. Tuberculosis-related mastitis was suspected. Anti-tuberculosis quadruple therapy (isoniazid, rifampicin, ethambutol, and pyrazinamide) was administered for 2 months, while isoniazid and rifampicin were administered for 7 months^2^ ^,^ ^3^. Post-treatment MRI showed no abscess or fistula tract formation (Figure 2). The 6-month follow-up revealed no new findings, demonstrating a complete response to tuberculosis mastitis treatment. It is important for tuberculosis to be considered in cases of mastitis, particularly when differentiating from widely seen idiopathic granulomatous mastitis.
